# Vertebral tuberculosis as a paradoxical reaction to the treatment of pulmonary and meningeal tuberculosis in an immunocompetent patient: A case report

**DOI:** 10.1097/MD.0000000000020012

**Published:** 2020-05-22

**Authors:** Cláudia Elizabeth Volpe-Chaves, Mara Luci Gonçalves Galiz Lacerda, Suse Barbosa Castilho, Simone Sousa Oliveira Fonseca, Bruna Abdul Ahad Saad, Caroline Franciscato, Tiago Kojun Tibana, Thiago Franchi Nunes, James Venturini, Sandra Maria do Valle Leone de Oliveira, Anamaria Mello Miranda Paniago

**Affiliations:** a Graduate Program in Infectious and Parasitic Diseases of Federal University of Mato Grosso do Sul; b Regional Hospital of Mato Grosso do Sul; c Maria Aparecida Pedrossian University Hospital; d School of Medicine at Federal University of Mato Grosso do Sul, Campo Grande, Mato Grosso do Sul, Brazil.

**Keywords:** extrapulmonary tuberculosis, meningeal tuberculosis, paradoxical reaction, Pott's disease, Tuberculosis, vertebral tuberculosis

## Abstract

**Introduction:**

Paradoxical reaction in tuberculosis (TB) is defined as the reappearance of general symptoms, aggravation of pre-existing diseases, or appearance of new lesions despite adequate anti-TB therapy. It may result from the hyperactivity of the immune response, resulting in an intense inflammation. There are few cases of vertebral TB reported as paradoxical reaction, mainly among immunocompetents patients.

**Patient concerns:**

We describe a male immunocompetent patient with confirmed pulmonary and meningeal TB. He was readmitted after 60 days of adequate treatment, with vertebral TB and paravertebral abscess, despite clinical improvement of the other locations. We defined as an uncommon case of a paradoxical reaction, confirmed by nuclear magnetic resonance and molecular rapid test for TB.

**Diagnosis:**

*Mycobacterium tuberculosis (*MTB) was detected in cerebrospinal fluid by molecular rapid test (Gene Xpert MTB/ rifampicina method). Sputum research and culture were positive for the same agent. Lumbosacral spine nuclear magnetic resonance revealed bone destruction from T8 to T11, and a paravertebral collection was found. Gene Xpert MTB/rifampicina and culture were positive for *M tuberculosis* in the drained material of the paravertebral abscess.

**Interventions:**

The paravertebral abscess was drainage by tomography-guided. Treatment with 4 anti-TB drugs was extended for 60 days and 2 anti-TB drugs was maintained for 10 months. There was a complete clinical improvement.

**Outcome:**

After draining the paravertebral abscess, the patient progressively improved and was discharged for outpatient follow-up. He was on antituberculous drugs for 1 year; subsequently, complete resolution of the infection was reported.

**Conclusion:**

Paradoxical reaction may be a difficult diagnosis in immunocompetent patient. Vertebral TB as a paradoxical reaction is an uncommon presentation. Therapeutic failure or resistance to treatment should be ruled out to confirm the diagnosis of paradoxical reaction.

## 1 Introduction

Paradoxical reaction in patients with tuberculosis (TB) is defined as the reappearance of general symptoms, aggravation of pre-existing diseases, or appearance of new lesions after an initial improvement, without a visible cause, at least 2 weeks from the start of treatment. It may result from the disinhibition of anti-TB cell-mediated immunity, resulting in an increased intensity of inflammation.^[1]^ High antigenic load and inflammation may act in association with pathogenesis and involve T-helper-1-induced immune response in the presence of multibacillary disease and immunodeficiency.^[2]^ With the control of TB, improved cellular immunity would be accompanied by an uncontrolled inflammatory reaction that exceeds the target involving specific adaptive immunity and innate immunity. Anemia, lymphopenia, hypoalbuminemia, and extrapulmonary localization can be risk factors in these patients.^[3]^

Pott's disease as a paradoxical reaction has been rarely reported, and the thoracic and lumbar spine are the most frequently described sites.^[3,4]^

In this study we describe a male immunocompetent patient with an uncommon case of vertebral TB as paradoxical reaction in confirmed pulmonary and meningeal TB despite adequate anti-TB therapy.

## 2 Case report

A 39-year-old male patient was admitted to the emergency room with anorexia, weakness, dry cough, weight loss and daily fever. He had a history of alcoholism. He presented normal laboratory tests, and his serologic test results were negative for HIV. Chest X-ray showed alveolar-interstitial consolidations in the upper lobes, with a suspected pulmonary TB (PTB).

During hospitalization, the patient had headache, mental confusion, stiff neck, and a positive Brudzinski sign. Skull computed tomography (CT) was normal, and the cerebrospinal fluid puncture presented 343 leukocytes/mm^3^; 95% lymphomononuclear cells; 5% polymorphonuclear cells; glucose, 25 mg/dL; protein, 435.6 mg/dL. *Mycobacterium tuberculosis* (MTB) without rifampicin (RIF) resistance was detected in cerebrospinal fluid by molecular rapid test (Gene Xpert MTB/RIF method). Sputum research and culture were positive for the same agent with sensitivity to the anti TB agents used, confirming the diagnosis of PTB and meningeal TB.

Four oral tablets (once daily) of 150-mg RIF, 75-mg isoniazid, 400-mg pyrazinamide, and 275-mg ethambutol, and 4-mg dexamethasone IV 6 /6 h (0.3 mg/kg/d) were administered and maintained until hospital discharge. The patient showed clinical improvement and was discharged on the 16th day of hospitalization. Prednisone was prescribed with doses reduction to suspension, with a total treatment time of <8 weeks, as observed in medical records.

On the 60th day of regular treatment with anti-TB drugs, although the patient reported improvements in pulmonary and meningeal manifestations, he returned to the hospital with fever and abdominal and lumbar pain. However, skull and chest CT and lumbar puncture evidenced no signs of disease activity.

**Figure 1:**
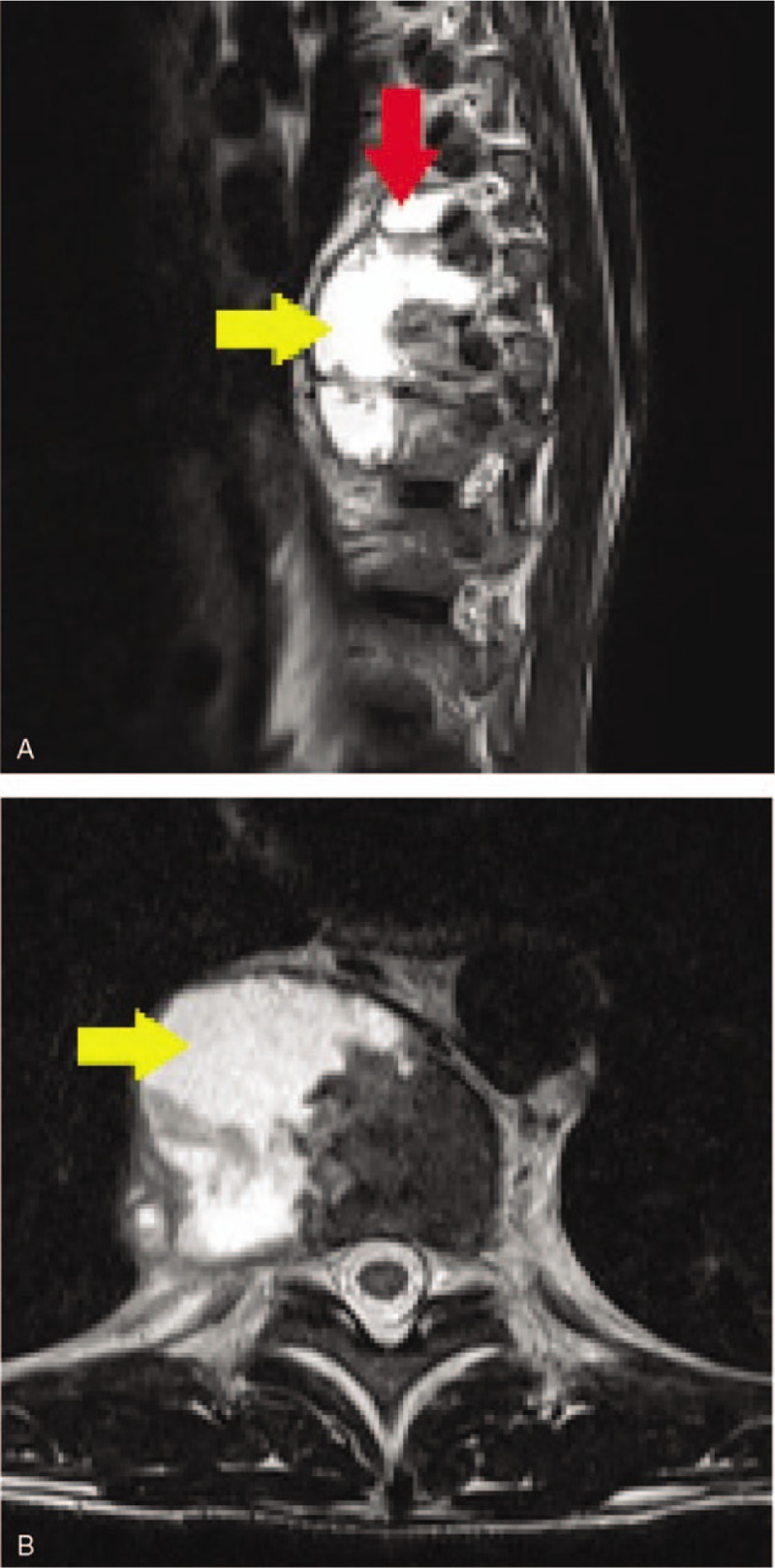
Lumbosacral spine nuclear magnetic resonance (NMR) of (A) the sagittal section and (B) the axial section. The NMR shows the extensive vertebral involvement of T8 to T11 (red arrow) and paravertebral abscess (yellow arrow). NMR = nuclear magnetic resonance.

Lumbosacral spine nuclear magnetic resonance revealed a signal alteration and a heterogeneous enhancement by contrast medium from T8 to T11, with reduction of the vertebral bodies’ height of T10 and T11 and collapse of the respective intervertebral space. A paravertebral collection was found, located adjacent to the vertebral bodies from T8 to T11, mainly on the right, measuring 7.0 × 4.8 × 3.0 cm, with an estimated volume of approximately 50 mL (Fig. 1).

Treatment with 4 anti-TB drugs was maintained, and morphine was prescribed due to severe pain. Tomography-guided abscess drainage was performed. Direct BAAR screening in the drained material was negative, but the Gene Xpert MTB/RIF and culture were positive for *M tuberculosis* with RIF sensitivity.

After this procedure, the patient showed clinical improvement and reduction of fever. The drain was removed after 10 days and a new control image revealed abscess reduction. The patient was discharged, and he received anti-TB drugs for 12 months from the date of abscess drainage. There was a complete clinical improvement, and the patient is under follow-up with a programmed neurosurgery for repairing a fracture by bone TB sequelae.

## 3 Discussion

Herein, we described an uncommon presentation of PTB and meningeal TB in an apparently immunocompetent patient. Upon completion of 60 days of effective anti-TB drug treatment with a marked improvement in pulmonary and meningeal focus, he developed vertebral TB and paravertebral abscess, both characteristics of a paradoxical reaction (PR).^[5]^

TB is classified into PTB, which includes cases of pulmonary impairment, pulmonary with extrapulmonary, laryngeal and concomitant miliary disease, as observed in our case, and extrapulmonary TB (EPTB), which involves any organs other than the lungs.^[6]^

Risk factors for PTB and EPTB are common, especially in patients with acquired immunodeficiency virus (HIV), diabetes, and malnutrition. Alcoholism seems to increase the risk for extrapulmonary locations, as observed in our report. Long-term corticosteroid and/or other immunosuppressive drugs were ruled out in our case. However, other causes, such as hereditary defects in cell-mediated immunity, were not investigated.^[7]^

Central nervous system involvement by *M tuberculosis* affects 0.5% to 2% of TB patients,^[8]^ with meningitis being the most severe form. In immunocompetent patients, TB meningitis constitutes 5% to 6% of EPTB cases and has a high morbidity and mortality rate,^[9]^ differing from the clinical improvement of our patient. Due to this severe form and in addition to the anti-TB therapy, corticotherapy was introduced, which is widely recommended in the literature.^[10]^

Osteoarticular TB accounts for 10% to 35% of extrapulmonary locations, and extension to adjacent soft tissues with cold abscess formation is commonly observed.^[7,11]^ The abscess formation is a strong indication for a potential diagnosis of TB,^[12]^ as it happened in our report.

The development of Pott's disease accounts for 40% to 50% of cases and it occurs due to hematogenous dissemination of the Batson venous plexus TB bacillus, secondary to an extravertebral source.^[12,13]^

PR was first described in non-HIV patients in 1954, after anti-TB drugs were introduced. ^[2]^ It is estimated to be present in 2% to 30% of immunocompetents patients,^[2,3]^ being more frequent in patients with extrapulmonary locations.^[2]^ PR is usually seen in patients with HIV, also called immune reconstitution syndrome, and it is poorly studied in immunocompetent patients.^[3,8,14]^

The PR usually observed in HIV-positive patients appears after starting HAART and is triggered by an excessive inflammatory response in the context of immune reconstitution.^[15]^ This immunity is not protective and, as a consequence, the existing disease may worsen or new lesions may appear.^[16]^ In HIV-negative patients showing PR to anti TB treatment (ATT), occurs as a result of ATT-induced disinhibition of cell-mediated immunity combined with the release of mycobacterial cell wall antigens during lysis of the microorganism.^[17]^

Our patient had no signs or symptoms of vertebral disease at admission that indicated MRI or thoracolumbar spine computed tomography. The emergence of new symptoms and new lesions despite adequate therapy after an initial clinical improvement, defined a PR in this case.

For the confirmed diagnosis of PR, we need to rule out differential diagnoses, such as inadequate anti TB therapy, and this item were ruled out in our report.^[14]^

The median time to onset of PR is 3 months after initiation of the anti-TB treatment in non-HIV-infected patients. In our report, this period was 2 months. Overall mortality in patients with PR has been estimated at 3%.^[2]^

In CNS infections, PR can occur in up to 52% of patients and has been reported as an important prognostic factor in young patients.^[5]^ Pott's disease as a paradoxical reaction has been rarely reported, and the thoracic and lumbar spine are the most frequently described sites,^[3,4]^ as noted in our report.

PR must be differentiated from therapeutic failure, and the anti-TB replacement is not indicated.^[3]^ In our report, we ruled out therapeutic failure based on the patient's report of regular use of therapy as well as on improvement in the signs and symptoms of pulmonary and meningeal TB diagnosed initially. In addition, the sputum culture described was sensitive to all anti TB agents. The patient responded satisfactorily with the same drugs for the treatment of spinal TB as for the PR.

In PR, the main interventions are related to complications, such as the need for surgical intervention and residual functional deficit.^[4,14]^ In our report, the first phase of TB treatment was extended for 60 days, and the use of corticosteroids—despite being considered^[4]^ —was not necessary.

The treatment of PR can be based on clinical and surgical interventions, and the use of steroids can be considered.^[18]^

Corticosteroids have been recommended in patients with tuberculous meningitis, especially for non-HIV patients,^[10,19]^ as used in our report, but there was a description of early reduction and suspension after hospital discharge. As PR is related to an intensification of the inflammatory response, we believe that this factor may have contributed to its manifestation soon after its suspension.

We report a rare case of vertebral TB as a PR in an immunocompetent patient. This diagnosis should be part of the clinical follow-up, especially in patients with extrapulmonary locations for TB, which may have a major impact on the patient's prognosis.

## Acknowledgments

The authors are grateful for the support of the Coordination for the Improvement of Higher Education Personnel - Brazil (CAPES).

## Author contributions

**Data curation:** Cláudia Elizabeth Volpe-Chaves, Mara Luci Gonçalves Galiz Lacerda, Suse Barbosa Castilho, Simone Sousa Oliveira Fonseca.

**Methodology:** Cláudia Elizabeth Volpe-Chaves, Mara Luci Gonçalves Galiz, Suse Barbosa Castilho, Simone Sousa Oliveira Fonseca, Bruna Abdul Ahad Saad, Caroline Franciscato, Tiago Kojun Tibana.

**Supervision:** Thiago Franchi Nunes, James Venturini, Sandra Maria do Valle Leone de Oliveira, Anamaria Mello Miranda Paniago.

**Writing – original draft:** Cláudia Elizabeth Volpe-Chaves, Mara Luci Gonçalves Galiz, Suse Barbosa Castilho, Caroline Franciscato, Simone Sousa Oliveira Fonseca, Bruna Abdul Ahad Saad, Tiago Kojun Tibana.

**Writing – review and editing:** Cláudia Elizabeth Volpe-Chaves, Thiago Franchi Nunes, James Venturini, Sandra Maria do Valle Leone de Oliveira, Anamaria Mello Miranda Paniago.
